# Risk Assessment of Genetically Engineered Maize Resistant to *Diabrotica* spp.: Influence on Above-Ground Arthropods in the Czech Republic

**DOI:** 10.1371/journal.pone.0130656

**Published:** 2015-06-17

**Authors:** Zdeňka Svobodová, Oxana Skoková Habuštová, William D. Hutchison, Hany M. Hussein, František Sehnal

**Affiliations:** 1 Faculty of Science, University of South Bohemia, České Budějovice, Czech Republic; 2 Institute of Entomology, Biology Centre CAS, České Budějovice, Czech Republic; 3 Department of Entomology, University of Minnesota, St. Paul, Minnesota, United States of America; French National Institute for Agricultural Research (INRA), FRANCE

## Abstract

Transgenic maize MON88017, expressing the Cry3Bb1 toxin from *Bacillus thuringiensis* (*Bt* maize), confers resistance to corn rootworms (*Diabrotica* spp.) and provides tolerance to the herbicide glyphosate. However, prior to commercialization, substantial assessment of potential effects on non-target organisms within agroecosystems is required. The MON88017 event was therefore evaluated under field conditions in Southern Bohemia in 2009–2011, to detect possible impacts on the above-ground arthropod species. The study compared MON88017, its near-isogenic non-*Bt* hybrid DK315 (treated or not treated with the soil insecticide Dursban 10G) and two non-*Bt* reference hybrids (KIPOUS and PR38N86). Each hybrid was grown on five 0.5 ha plots distributed in a 14-ha field with a Latin square design. Semiquantitative ELISA was used to verify Cry3Bb1 toxin levels in the *Bt* maize. The species spectrum of non-target invertebrates changed during seasons and was affected by weather conditions. The thrips *Frankliniella occidentalis* was the most abundant species in all three successive years. The next most common species were aphids *Rhopalosiphum padi* and *Metopolophium dirhodum*. Frequently observed predators included *Orius* spp. and several species within the *Coccinellidae*. Throughout the three-year study, analysis of variance indicated some significant differences (P<0.05). Multivariate analysis showed that the abundance and diversity of plant dwelling insects was similar in maize with the same genetic background, for both *Bt* (MON88017) and non-*Bt* (DK315) untreated or insecticide treated. KIPOUS and PR38N86 showed some differences in species abundance relative to the *Bt* maize and its near-isogenic hybrid. However, the effect of management regime on arthropod community was insignificant and accounted only for a negligible portion of the variability.

## Introduction

Transgenic maize expressing insecticidal toxins such as Cry1Ab or Cry3Bb1, will likely be an important novel pest management technology in Europe because chemical control for lepidopteran and coleopteran pest species is more expensive, generally provides inconsistent efficiency and poses environmental risks [1]. The recent threat of the highly adaptive invasive pest, the western corn rootworm (WCR), *Diabrotica virgifera virgifera* LeConte (Coleoptera: Chrysomelidae), has become a particularly significant concern, given its continued spread in Europe. This species is one of the most destructive insect pests of maize in the U.S. [2]. Following the detection of *D*. *v*. *virgifera* in Serbia in 1992, the pest has expanded its range to several other European countries [3], including the Czech Republic in 2002 [4].

The insecticidal spectrum of Cry3Bb1, derived from *Bacillus thuringiensis* (*Bt*) subsp. *kumamotoensis*, includes larvae of the Colorado potato beetle (*Leptinotarsa decemlineata)* and the corn rootworm species (*Diabrotica* spp.) [5,6]. Previous research on Cry3Bb1 has shown that it was effective in reducing the number of *D*. *v*. *virgifera* larvae under field conditions [7]. However, larval mortality was rarely 100%, and the first report on *D*. *v*. *virgifera* resistance in field populations in the central U.S. has been published [8]. Another concern with Cry3Bb1 maize is the possibility of variable *Bt* expression under different environmental conditions [9].

Cry3Bb1 expressed in MON88017 was modified to increase efficiency. It is different from the original *Bt* toxin in six amino acid positions, and from the Cry3Bb1 in MON863 in a single amino acid position [9]. The MON863 event was approved for commercial use in the United States in 2003, and MON88017 in 2005. Both events are registered and authorized in the EU as genetically modified food and feed but not for planting. In the EU, MON863 is currently in the renewal stage of authorization as a food and feed, while the expiration date for MON88017 is October 2019 [10]. The process to approve Cry3Bb1 maize for the commercial cultivation in the EU is still ongoing, even though the dossier was submitted in 2008 [1]. Prior to approval, extensive field studies are generally required and should be performed to evaluate *Bt* maize in European agroecosystems and under EU production practices.

Laboratory studies designed to assess potential negative effects of Cry3Bb1 have been previously conducted for plant dwelling arthropods or for predators living on the soil surface [11–21]. Although Cry3Bb1 was detected in non-target phytophagous and predatory species [18–22], no detrimental effects on their development or reproduction were observed. Such studies were conducted in many countries, including several European laboratories [18–21]. On the other hand, large-scale and long-term field trials focusing on the comparison of transgenic plants with near-isogenic hybrids, and with conventional pest management have declined sharply in the EU since 1999 [23]. The setback of research in many EU countries might be attributed to the numerous bureaucratic requirements for the conduct of biosafety testing and to the risk of vandalism of field experiments with GM crops [24]. In some cases, negative public opinion about GM crops [25] can also play a role in authorization despite a high adoption rate among growers in many countries worldwide [26], and a growing number of significant cumulative benefits from planting GM crops [26–30].

Given the concerns regarding potential non-target impacts of GM maize in the Czech Republic, the purpose of our study was to examine the extent to which the abundance of non-target, above-ground arthropods might be affected by *Bt* maize expressing Cry3Bb1 under field conditions. We compared arthropod abundance throughout three growing seasons for Cry3Bb1 *Bt* maize its near-isogenic hybrid and two reference non-*Bt* hybrids. We also tested the effect of soil insecticide chlorpyrifos applied as a preventive measure against *D*. *v*. *virgifera*. We confirmed that soil application of insecticide at planting does not affect plant dwelling species substantially [31]. We also verified the statement that differences between hybrids are more pronounced than the effect of transgene expression [22].

## Materials and Methods

### Experimental design and agricultural management

The Ministry of the Environment of the Czech Republic approved this study (permission No. 78379/ENV/08 issued on January 12, 2009, European notification number B/CZ/08/03). The study was performed in 2009–2011 on 25 plots in Southern Bohemia in the vicinity of České Budějovice on part of the privately owned land block 6201/1 cultivated by the Agricultural Company Dubné a.s. The average annual temperature was about 11°C and the average annual precipitation 550 mm. Temperature and precipitation were measured 13 km away from the field by the Hydrometeorological Institute in České Budějovice.

A 14-ha rectangular field was divided with the aid of GPS into twenty five 0.5 ha (63 x 81 m) plots arranged in 5 columns (63 m wide) and 5 rows (81 m wide) separated by 2–3 m strips of bare land (see [32]). One of the shorter sides of the rectangle was adjacent to a forest; the other sides were flanked by wheat fields. The Latin square design was slightly disturbed by the presence of drainage wells in nine plots and a hunting lookout in one plot. Genetically engineered *Bt* and herbicide tolerant maize, MON88017 (YieldGard VT Rootworm/RR2, MONSANTO Technology LLC, St. Louis, MO, USA), its near-isogenic hybrid DK315 untreated or treated with the wide-spectrum soil insecticide Dursban 10G (a.i. chlorpyrifos, 20 kg/ha), and the conventional reference hybrids KIPOUS (KWS SAAT AG, Einbeck, Germany) and PR38N86 (DuPont Pioneer, Johnston, IA, USA) were each planted in 5 randomly distributed plots. Insecticide was applied simultaneously with maize seed sowing. Field margins were seeded with the early maize hybrid DKC2870 (MONSANTO Technology LLC, St. Louis, MO, USA).

Planting commenced when the average day temperature reached 10°C on May 11 in 2009, and May 6 in 2011. In 2010, planting was postponed to June 10 (25°C) because of inclement weather conditions unsuitable for sowing. Standard treatments with pre- and postemergence herbicides and a fertilizer were used (see [32]). In 2009, plants in the waxy stage of kernel ripening (dough stage, BBCH 85, [33]) were shredded to pieces smaller than 1 cm and ploughed into the soil on the same plot where the respective maize hybrid had been grown. In the following two years, all experimental plots including field margins were harvested and used for biogas production. Remains after biogas production (digestate) were ploughed into the field. Each plot was planted with the same maize hybrid in all three successive years.

### Monitoring of non-target organisms

The presence of plant dwelling insects was recorded at the leaf stage development BBCH 14 (four leaves unfolded), BBCH 16 (six leaves unfolded), BBCH 65 (middle of flowering) and BBCH 85 (dough stage). The sampling at BBCH 14 was omitted in 2009 due to exceptionally rainy June which made sample collection impossible. On each sample date, 10 randomly selected plants per plot were individually covered with sacs made from synthetic fabric (available in three sizes) and closed tightly. This method was successfully used in previous field experiments [34,35]. Two to three plants randomly chosen in each plot were taken with the root system and inspected for the presence of root pests. Plants were transported to the laboratory and visually inspected on the same day to estimate the presence of arthropods. The sacs with plants were held in a cold room (4°C) and taken out just before insect collection for identification and counting. Insect mobility was substantially reduced by chilling and most specimens dropped to the sack bottom.

Adult parasitoids and predators were monitored by using yellow sticky traps (Bio Plantella, Unichem d.o.o., Slovenia). Two pieces of two-sided sticky traps (240 x 180 mm) were placed on plants at a height of 1.8 m in the centre of each plot in the middle of July and first half of August at BBCH 53 (heading stage) and BBCH 65, for a total of 14 days at each growth stage.

### Monitoring of the target species, *Diabrotica virgifera virgifera*

The occurrence of *D*. *v*. *virgifera* was monitored with 10 pheromone traps (Csalomon PAL, Plant Protection Institute, Centre for Agricultural Research, Hungarian Academy of Sciences, Hungary) which were placed on plants at the margins of the experimental area in early July and removed just before harvest. The traps with synthetic sex pheromone attract males and are recommended for *D*. *v*. *virgifera* monitoring [36].

### Toxin measurements

The amount of Cry3Bb1 toxin was measured in maize (roots, aerial roots, stem and leaves) at the time of arthropod collections (three times in 2009 and four times in 2010 and 2011). Samples of pollen, tassels and stigmas were taken once per season (BBCH 65) and samples of grain were taken twice per season (BBCH 65, BBCH 85). One sample was taken from each *Bt* maize plot at each sample date, from all other plots twice per year (mix sample per management regime), in maize stage BBCH 14 (BBCH 16 in 2009) and BBCH 85. The samples were stored at −20°C for not more than 60 days before analysis. No reduction in the level of Cry3Bb1 toxin in *Bt* maize pollen stored at −20°C for 49 days, was reported [11]. At the beginning of our study we confirmed the stability of toxin content in maize tissues stored at −20°C for 60 days (data not shown).

Toxin content in collected samples was measured with the commercial *Bt*-Cry3Bb1 ELISA PathoScreen kit (Agdia Inc., Elkhart, IN, USA). For all plant samples, 1 g tissue was homogenized in a Homex device (Bioreba AG, Switzerland) in 10 ml of the extraction buffer (PBST) provided with the kit. The mash was centrifuged (Hettrich Zentrifugen, EBA 12R) at 8 080 x g for 5 min, at 4°C. The supernatant was diluted differentially because we assumed changes in the Cry3Bb1 content during maize development [9]. Samples from the BBCH 14 growth stages were diluted 1:8 000 and those from the BBCH 16, 65 and 85 stages at 1:25 000–50 000. Samples were analysed according to the manufacturer’s protocol.

Positive control sample provided by Agdia Inc. contained approximately 40 ng/ml Cry3Bb1 and enabled semiquantitative measurements. A two-fold dilution series ranging from 0.3125 to 40 ng/ml was prepared to construct a standard curve. Absorbance was determined by ELISA reader (Spectra MAX 340 PC, λ = 650 nm). GraphPad Prism 5 (GraphPad Software Inc., San Diego, CA, USA) was used for interpolation of the concentration from the standard curve. The limit of detection was determined as a triple of the standard deviation of the control value.

### Statistical analyses

GraphPad Prism 5 was used for descriptive statistics and Statistica 8 (StatSoft Inc., Tulsa, OK, USA) for the analysis of variance (Factorial ANOVA, data from sample dates were summed, factors: year and management regime) of arthropod abundance (log transformation of data recorded from sticky traps). The post-hoc Tukey HSD tests were also processed in Statistica 8. Concentration of Cry3Bb1 toxin in plant tissues was compared using Main effects ANOVA (factors: year, sample date, plant tissue).

The ecological preferences were assessed in CANOCO for Windows 4.5 (Biometris—Plant Research International, Wageningen, The Netherlands). Linear character of arthropods community was confirmed by detrended correspondence analysis (DCA: detrending by segments, log transformation, downweight rare species). The Monte Carlo permutation test, MCPT (log transformed species data, 999 permutations, forward selection) with environmental variables: time series (number of days since the sowing day marked as number 1), year, maize hybrid, plot position within the field and plot disturbance by the presence of the drainage wells or hunting lookout were employed in the multivariate redundancy data analysis (RDA) with plant numbers or sticky traps as covariates. RDA was in partial shape (covariate’s influence was subtracted) and split-plot design [37]. Variables describing geographical disposition were tested to verify the independence of species abundance on these environmental variables. 5% level of significance was considered in all statistical tests.

## Results

### Community of plant dwelling non-target arthropods

**Fig 1:**
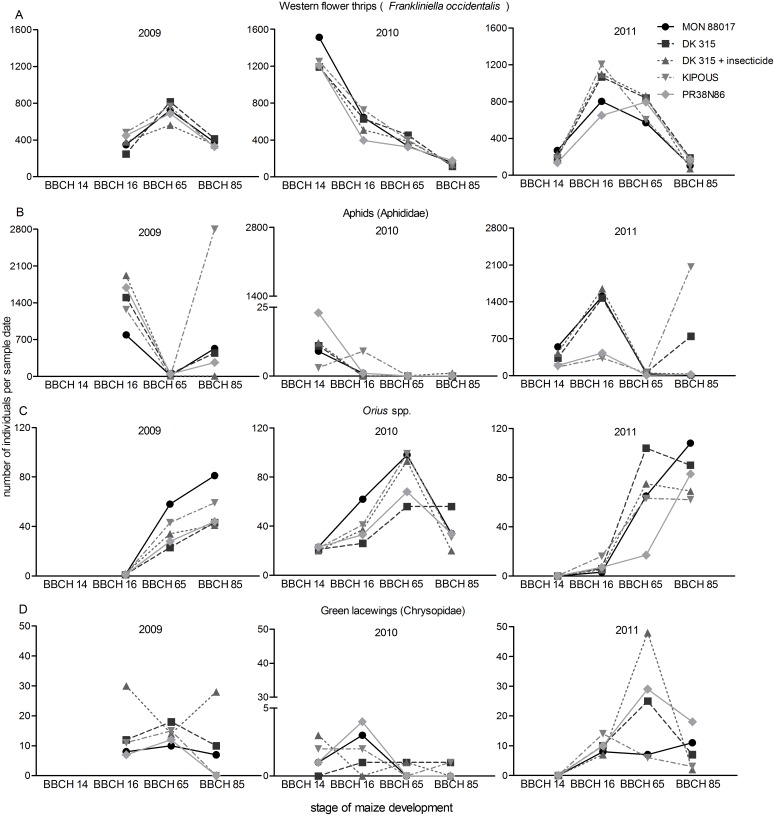
Seasonal fluctuations of common plant dwelling insects in the trial years. Number of individuals per sample date (50 plants per management regime). A: Western flower thrips (*Frankliniella occidentalis*). B: Aphids (Aphididae: *Metopolophium dirhodum*, *Rhopalosiphum padi*, *Sitobion avenae*). C: *Orius* spp. D: Green lacewings (Chrysopidae)

Nine different orders of arthropods were recorded on all maize hybrids, including *Bt* maize (Table 1, numbers of individuals per plant are provided in S1 Table). The plant-sucking western flower thrips (*Frankliniella occidentalis*) was common in all plots, with highest abundance at the BBCH 65 stage or before (Fig 1). Approximately 15, 25 and 19 thrips per plant were found in the midsummer in 2009, 2010 and 2011 respectively. The lowest abundance of *F*. *occidentalis* was on reference hybrid PR38N86, but the differences were not statistically significant (Table 1).

**Table 1:** Numbers (mean per management regime and year ± SD) of arthropod counts (management regimes are explained in Material and Methods) in three consecutive years (2009–2011), and the results of the analysis of variance for differences in arthropod abundance among plots with different management regimes.

			Management Regime					
Order	Family	Species	MON88017	DK315	DK315 + insecticide	KIPOUS	PR38N86	ANOVA (df = 4,60)
Araneae	unidentified		44.3±14.0	50.7±23.6	41.3±12.3	45.7±6.3	36.7±9.4	F = 0.6, P = 0.68
Coleoptera	Coccinellidae	unidentified	28.7±11.1	31.0±9.4	16.3±5.0	15.7±5.9	27.7±11.1	F = 0.6, P = 0.64
	Elateridae	unidentified	0.3±0.5	0	9.0±12.7	0.3±0.5	2.7±3.8	F = 1.6, P = 0.19
	Staphylinidae	unidentified	6.7±1.7	5.7±2.5	5.3±2.1	4.0±1.4	9.0±5.9	F = 1.1, P = 0.37
	unidentified		15.3±4.2	14.0±3.6	11.7±4.5	4.3±0.9	11.0±6.5	F = 1.8, P = 0.13
Diptera	Syrphidae	unidentified	28.0±11.0	22.3±7.8	23.3±3.4	17.0±9.9	29.0±17.0	F = 1.6, P = 0.18
Hemiptera	Anthocoridae	*Orius* spp.	176.7±34.0a	132.7±56.1ab	143.3±42.9ab	152.3±37.4ab	111.0±35.3b	F = 2.9, P = 0.03
	Aphididae	*Metopolophium dirhodum*	479.0±455.5	449.7±448.7	517.7±408.8	834.7±962.3	207.3±141.9	F = 0.6, P = 0.67
		*Rhopalosiphum padi*	674.3±475.4	1086.3±769.2	885.3±639.4	1397.3±1677.7	698.7±771.4	F = 1.3, P = 0.28
		*Sitobion avenae*	0.3±0.5	2.3±3.3	0.7±0.9	0	0.7±0.9	–*
	Miridae	unidentified	2.7±2.5	2.3±2.1	1.3±0.9	4.0±2.9	2.7±2.5	–
	Psyllidae	unidentified	1.3±1.9	2.0±2.2	1.7±2.4	1.3±0.9	2.3±2.1	–
Hymenoptera	Braconidae	unidentified	6.0±1.4	7.7±3.4	2.3±0.9	6.3±4.2	6.7±4.6	F = 1.2, P = 0.31
Lepidoptera	Noctuidae	*Helicoverpa armigera*	0	0	0.3±0.5	0	0	–
	Crambidae	*Ostrinia nubilalis*	10.7±11.1	9.3±11.1	22.0±12.4	16.7±19.3	22.3±22.4	F = 1.7, P = 0.16
Neuroptera	Chrysopidae	unidentified	18.3±10.1a	28.7±18.2ab	43.7±28.5b	18.7±9.9a	27.0±22.0ab	F = 3.0, P = 0.03
Prostigmata (Acari)	unidentified		2.3±3.3	1.0±0.8	3.3±2.4	1.3±1.9	1.7±2.4	–
Thysanoptera	Aeolothripidae	*Aeolothrips fasciatus*	4.7±3.9	5.3±3.3	2.0±2.8	1.0±1.4	2.3±3.3	F = 1.6, P = 0.19
	Thripidae	*Frankliniella occidentalis*	1943.7±504.5	2065.7±386.7	1929.0±425.6	2059.0±373.1	1773.7±264.4	F = 0.6, P = 0.64

Different letters denote significant differences (post-hoc Tukey HSD test).

*–, not enough variance for test.

The abundance of aphids (Aphididae) was high in 2009 and 2011 (Fig 1). Aphids were predominantly represented by the bird cherry-oat aphid (*Rhopalosiphum padi*) and the rose-grain aphid (*Metopolophium dirhodum*). Both species reached their highest numbers in maize stage BBCH 16 (29 and 22 individuals per plant in 2009 and 2011, respectively (Fig 1). The abundance of *M*. *dirhodum* was similar on MON88017 and its near-isogenic hybrid, untreated or treated with insecticide, and was slightly different from both reference hybrids, albeit insignificantly (Table 1). *R*. *padi* was most abundant on the reference hybrid KIPOUS but differences from other hybrids were not significant (Table 1). Interaction between year and management regime was significant (F(8,60) = 2.7, P = 0.01) because more individuals of *R*. *padi* on the reference hybrid KIPOUS were recorded only in 2009.

The 3^rd^ collected aphid species, *Sitobion avenae*, was not as common as *M*. *dirhodum* and *R*. *padi*. Its abundance was similar on plots with different management regimes. Interaction was significant (F(8,60) = 2.2, P = 0.04) because number of *S*. *avenae* was higher on the near-isogenic hybrid DK315 in 2009.

Eggs and larvae of the European corn borer (ECB, *Ostrinia nubilalis*) were detected in all plots (Table 1); in not significantly lower numbers on MON88017 and the near-isogenic hybrid DK315.

Plant dwelling predators included several generalists, of which *Orius* spp. were most common (Table 1). Densities of *Orius* spp. peaked in maize stage BBCH 65 and even later in stage BBCH 85 in 2011 (Fig 1). The abundance of *Orius* spp. was highest on MON88017—significantly higher than on the reference hybrid PR38N86 (Table 1).

Many adults of lady beetles (Coccinellidae) and green lacewings (Chrysopidae) flew away during collection of the plant samples. Nevertheless, a high proportion of the Coccinellidae counts consisted of adults. Their numbers decreased during the season. Very few larvae were found despite the fact that wooden marker sticks placed at the edges of plots harbored high numbers of Coccinellidae pupae, particularly in 2011. Most counts of green lacewings were limited to the egg stage. The highest number of individuals was found on near-isogenic hybrid DK315 treated with insecticide—it was significantly higher than on MON88017 and the reference hybrid KIPOUS. Results of separate statistical analysis of predators and other monitored arthropods are listed in Table 1. Besides the above mentioned significant differences the abundance of plant dwelling non-target arthropods was similar on plots with different management regimes. Interaction was significant for larval Syrphidae (F(8,60) = 2.1, P = 0.05) that occurred in highest number on the reference hybrid PR38N86 in 2009.

**Fig 2:**
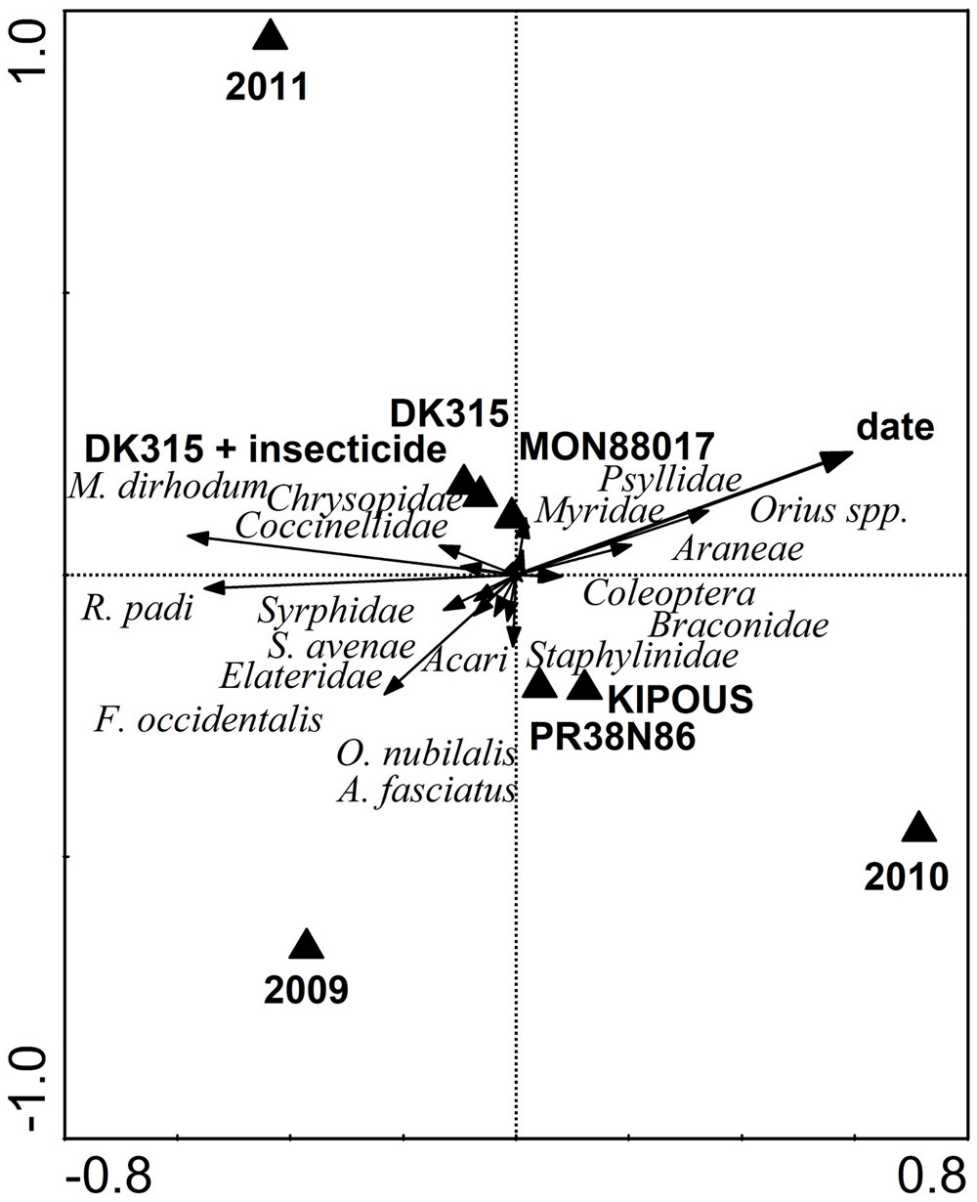
Redundancy analysis of the spatial and temporal distribution of plant dwelling arthropods. Horizontal line indicates years and sample date and vertical line the management regimes.

Confirmation of the linear character of arthropod community (DCA: length of gradient: 2.8) was followed by integrated analysis of ecological preferences of plant dwelling arthropods implemented using a partial RDA. From the percentage of explained variability it is clear that arthropods did not prefer or avoided any specific *Bt* or non-*Bt* management regime (Table 2). However, two separate clusters of centroids for management regimes in Fig 2 show that the abundance of plant dwelling insects was similar in the genetically related hybrids—MON88017 and DK315, untreated or treated with insecticide. The reference hybrids KIPOUS and PR38N86 were somewhat different from the *Bt* maize and its near-isogenic hybrid, but similar to each other. The total contribution of management regimes to the explained variability was small (Table 2). The cluster of centroids near the intersection of the ordination space (crossing of the first and second axis) confirms low explanatory power of management regimes. Many other factors which could influence arthropod distributions in the field were rejected as well (plot position within the field, plot disturbance by the presence of the drainage wells or hunting lookout). Most of the variability in arthropod distributions was explained by the sample date and year.

**Table 2:** Results of Monte Carlo permutation tests (MCPT) in RDA analyses.

MCPT	% variability explained	
Environmental variables	Visual inspection	Sticky traps
MON88017	0.1	1.4
DK315	0.1	0.5
DK315 + insecticide	0.1	0.9
KIPOUS	0.2	0.8
PR38N86	0.1	1.6
Year	9.4**	19.6**
sample date	5.9**	7.0**
row	0.1	3.6
column	0.0	0.4
well	0.1	0.7
hunting lookout	0.1	1.3

Asterisks denote significant differences (** P<0.01).

### Monitoring of parasitoids and predators on yellow sticky traps

Representatives of five insect orders of natural enemies were recorded on the sticky traps (Table 3, numbers of individuals per sticky trap are provided in S2 Table). The fly family Syrphidae, with the dominating marmalade hoverfly (*Episyrphus balteatus*), was particularly common. *Scaeva pyrastri* and two *Sphaerophoria* species were recorded less frequently. Although syrphid numbers were low in 2010 with 262 adults, 2139 and 3022 individuals were trapped in 2009 and 2011, respectively. Statistical analysis of abundance revealed significantly higher numbers of Syrphidae on the near-isogenic hybrid DK315 than on the reference hybrids KIPOUS.

**Table 3:** Numbers (mean per management regime and year ± SD) of predators and parasitoids collected in the sticky traps during the growing season in each trial year (2009–2011), and the results of analysis of variance for differences in abundance among plots with diverse management regimes (management regimes are explained in Material and Methods).

		Management Regime					
Order	Family*	MON88017	DK315	DK315 + insecticide	KIPOUS	PR38N86	ANOVA (df = 4,60)
Coleoptera	Coccinellidae	24.7±20.1	13.7±5.9	14.7±13.7	14.3±8.3	24.7±21.5	F = 1.5, P = 0.21
Diptera	Syrphidae	378.3±243.1ab	444.3±252.2a	330.3±199.1ab	304.3±219.0b	344.3±254.3ab	F = 2.7, P = 0.04
Hymenoptera	Braconidae and Ichneumonidae	53.7±33.6	32.7±26.6	50.0±35.3	21.3±17.0	25.3±30.2	F = 1.3, P = 0.26
Neuroptera	Chrysopidae and Hemerobiidae	5.7±5.2	7.7±9.4	8.3±5.2	12.3±5.6	7.7±5.9	F = 1.4, P = 0.25
Mecoptera	Panorpidae	3.7±0.9ab	8.7±5.0ab	3.3±3.3a	7.7±8.1ab	13.7±8.3b	F = 2.5, P = 0.05

Different letters denote significant differences (post-hoc Tukey HSD test).

*species specified in Results.

Only 20 adult parasitoids (Ichneumonidae and Braconidae) were collected in 2009. In subsequent years, the number of captured specimens increased to 198 individuals in 2010 and to 331 in 2011. Similar to the parasitoids and Syrphidae, aphid predators *Chrysoperla carnea* (Chrysopidae) and *Micromus variegatus* (Hemerobiidae) were found more frequently in 2011 than in 2009 and 2010 (including visual sampling, Fig 1). Lady beetles (Coccinellidae) were collected in low numbers in 2010 and 2011. *Propylaea quatuordecimpunctata* was the most common lady beetle found on sticky traps, followed by the invasive *Harmonia axyridis*. *Coccinella septempunctata*, generally the most common Czech lady beetle, was recorded only sporadically, similarly to *Adalia bipunctata*, *Coccinella quinquepunctata* and *Hippodamia variegata*. The scorpion fly *Panorpa communis* (Panorpidae) was less abundant on the near-isogenic hybrid DK315 with insecticide than on the reference hybrid PR38N86. Abundance of other arthropods was similar on all plots, irrespective of the management regime (Table 3) and interaction was also insignificant.

The partial RDA (DCA: length of gradient: 1.7) indicated slightly higher, but also insignificant, explanatory power of management regimes than was found in the previous analyses (Table 2). The comparison is approximate because arthropod communities were grossly disproportionate for both abundance and the number of detected taxa.

### Monitoring of *D*. *v*. *virgifera*

No *D*. *v*. *virgifera* was detected with the use of pheromone traps in any of the three years. No other evidence of *D*. *v*. *virgifera* presence, such as larval feeding damage on roots or adult feeding on stigmas or silk tissue was found.

### Concentration of Cry3Bb1 toxin in plant tissues

The content of toxin in different parts of the *Bt* maize plants fluctuated around 25 μg per 1 gram fresh plant tissue. Highest expression of Cry3Bb1 toxin was recorded in the leaves (36.28 μg g^-1^) and roots (30.74 μg g^-1^). Average amounts of Cry3Bb1 toxin determined in all three years are shown in Table 4. Level of Cry3Bb1 expression in different parts of maize plants during seasons 2009–2011 is provided in (S3, S4 and S5 Tables). Small standard deviations indicate that the differences between samples (growth stage) were minimal (F(3,218) = 0.49, P = 0.69) but differences between years were recorded (F(2,218) = 11.7, P<10^–4^). The limit of detection was estimated to be 0.08 ng per 1 g of fresh tissue. No toxin was detected in the near-isogenic and in the reference hybrids.

**Table 4:** Content of Cry3Bb1 determined by ELISA in indicated tissues of MON88017 maize grown in the field in 2009–2011. Mean (± SD) value per sample (growth stage).

	Mean content of Cry3Bb1 (μg per g of fresh material) in maize MON88017		
Maize tissues analyzed	2009	2010	2011
root	30.74±0.67	23.35±1.40	23.65±1.02
aerial root	18.68±0.77	nd^a^	nd
stem	11.90±0.24	22.48±2.13	20.87±1.38
leaf	36.28±0.15	28.60±1.60	36.06±1.08
pollen + tassels	28.20±1.40	20.94±1.49	15.01±2.34
stigmas	27.82±0.34	14.93±1.69	10.46±1.56
grain	nd	7.70±0.29	20.00±2.11

Concentration of Cry3Bb1 in pollen, tassels and stigmas was measured only once per season and twice in grain.

^a^ nd, not determined

## Discussion

The range of taxonomic groups of phytophagous insects and their predators found in this study fits with records available for maize in the database of non-target arthropod species occurring in arable crops across Europe [38]. Our data are also similar to the results of trials conducted in the U.S. [14,31,39]. The consistence of our findings with the results of other studies, especially in Europe, could be important for developing an appropriate post market monitoring plan, as required by EU legislation [40].

Although we performed exhausting monitoring of the above-ground arthropods, we did not include assemblages of insects active at night. Other collection methods would be needed for such studies. The use of light traps that are a usual tool for night insect trapping, is probably not suitable for manipulative experiment because it is unlikely that light traps would attract insects separately from each plot.

Two significant differences in the abundance of arthropods (*Orius* spp., Syrphidae) were found between maize hybrids. Consistent and significant influence of the hybrid type was previously found in the field densities of the herbivorous plant bug *Trigonotylus caelestialium* [22]. We were unable to assess possible hybrid influence on the bug abundance because we failed to capture sufficient number of specimens for the analysis of variance. It is possible that the population density of Miridae bugs was low in our field but we cannot exclude that their numbers were underestimated due to the sampling methodology we used. The counts of spiders (Araneae), adult lady beetles (Coccinellidae) and green lacewings (Chrysopidae) were probably also undervalued. We even did not find any leafhoppers (Cicadellidae) and adult Syrphidae. We are aware of the shortcomings of our methodology [34] but it is nevertheless suitable and effective for sampling herbivores that are in direct contact with plant tissue expressing the insecticidal toxin. Supplementation of this method with suitable sticky traps allows monitoring of flying herbivores, predators, parasitoids and pollinators. Aphids accounted for ca. 40% of all insects collected in this study. The size of aphid populations varied between the plots, probably due to aphid mobility and random plant colonization followed by rapid population growth. No effect of *Bt* hybrid on aphid abundance was observed in our study, consistently with previous investigations [31]. Also, a review article concluded that there are no significant effects of *Bt* maize on aphids [41] and that the risk of *Bt* maize to aphids and their predators is negligible.

Approximately 50% of all insect specimens found on each maize plant consisted of *F*. *occidentalis*. Thrips abundance on MON88017 was not different from other management regimes. Similar thrips insensitivity was documented for *Bt* maize expressing Cry1Ab [34]. We are not aware of any previous publications addressing the effect of Cry3Bb1 *Bt* maize on *F*. *occidentalis*.

According to the statistical analysis of our data, the presence of Cry3Bb1 had no effect on the abundance of predators and parasitoids except for *Orius* spp. and Chrysopidae, whose abundance was actually highest and lowest, respectively, on the *Bt* hybrid MON88017. Higher abundance of *Orius* spp. in *Bt* maize was previously recorded [42,43]. However, lack of effect was found in meta-analysis [44] that included data from 13 field trials in Spain, one of them with higher abundance of *Orius* spp. in *Bt* maize. It was suggested that higher *Orius* spp. densities in *Bt* maize could be related to their preference for ears and silks free from lepidopteran feeding [42,43]. It is not satisfactory explanation in case of maize resistant to corn rootworms. A laboratory study found increased fecundity and shorter nymphal development of *Orius majusculus* feeding on diets (leaves, pollen, *Tetranychus urticae*) containing Cry1Ab [45], suggesting that the occasionally observed higher abundance of *Orius* spp. in *Bt* maize could be due to unidentified differences in food properties. Laboratory assessment of *Orius* spp. response to *Bt* maize expressing other Cry toxins could help to solve this puzzle. Significantly less green lacewings (Chrysopidae) were found on sticky traps in the genetically modified Roundup Ready soybean than in the standard soybean cultivars [42]. However, other authors [14,31,39] did not find any substantial difference in the visual counts of *Orius insidiosus* and *C*. *carnea* [39] on the Cry3Bb1 maize and its near-isogenic hybrid.

Numerous studies focused on Coccinellidae because they include key predatory species used for biological control of various pests worldwide [46] and because beetles are the target of Cry3Bb1-expressing *Bt* crop. Several precise tritrophic experiments providing worst-case exposure conditions did not reveal any negative effect of purified Cry toxins and *Bt* maize on Coccinellidae (for example [20,21]). Exposure to the Cry toxins in the field is generally low for lady beetles that feed aphids as their major food source because aphids contain only trace amounts of Cry toxins [41]. None of the field trials conducted with Cry3Bb1 *Bt* maize disclosed an effect on Coccinellidae [14,31,39,47]. However, there are specialist predators of spider mites, containing high level of Cry toxins, such as *Stethorus punctillum*. Negative effect of Cry3Bb1 on *S*. *punctillum* fed to *T*. *urticae* was not confirmed—on the contrary, female beetles had a shorter pre-oviposition period and increased fecundity and fertility [20]. It should be mentioned that *S*. *punctillum* is not on the list of species found in above mentioned field trials. We also did not find this species on the sticky traps.

Parasitoid numbers caught by the sticky traps were relatively low in our study, particularly in the first year. Previous laboratory studies had demonstrated safety of Cry1Ab, Cry1Ac and Cry3B to Braconidae [48,49], Cry1C to Ichneumonidae [50] and Cry9Aa to Tachinidae (Diptera) [51]. However, the need to perform tests in a more realistic scenario was emphasized [51]. In previous studies, MON810 maize did not alter the aphid—parasitoid ratio and had no effect on aphid parasitism in the field [52]. No effect on braconid abundance was found in maize expressing Cry3Bb1 [38]. Results of several other studies were reviewed and meta-analysed to show no significant reduction of parasitoids not specialized for target pests of *Bt* maize [28,53].

Overall catches of adult predators were also low, except for Syrphidae. The decrease in their abundance in 2010 could be due to the low occurrence of aphids, common prey of their larvae, in that year—the nectar-feeding adults efficiently locate and lay their eggs close to aphid colonies [54]. The density of Syrphidae was independent of Cry3Bb1 expression, similar to previous study [31].

The soil treatment with a non-systemic insecticide performed in our study did not affect species abundance, except for significantly lower numbers of *P*. *communis* by comparison with the reference hybrid PR38N86. The abundance of Chrysopidae monitored by visual samplings was highest on the near-isogenic hybrid DK315 treated with the insecticide but this was not confirmed by insect samplings with the sticky traps. Insecticide applications to soil usually exert damaging effect on the ground-dwelling arthropods [55]. Our study shows that soil insecticide applied during maize sowing does not have negative effects on the plant dwelling arthropods, although the lower abundance of *P*. *communis* should be verified.

The content of Cry3Bb1 in transgenic plants depends on many variables including climate and weather conditions, maize genotype, tissue and growth phase; measurements are also affected by the extraction method [9,56]. These factors account for high variability of detected amounts of Cry3Bb1 in different studies [9,14,20,57]. Young tissue contained more Cry3Bb1 than the old tissue and significant differences in the level of expression among years were detected [9]. We did not find high variation during the season, but the variation between years was confirmed.

The region infested with *D*. *v*. *virgifera* in the Czech Republic continues to expand. The species was found 90 km away from our plots in 2010 and only within 35 km in 2011 [58]. *Diabrotica v*. *virgifera* is an invasive pest coming from outside Europe without specialized predators or parasitoids; biological control agents are also lacking in North America [59]. In the newly occupied territories, *D*. *v*. *virgifera* is apparently consumed by general predators but there is no evidence for an increase of their population density [31]. Hence, there is no reason to believe that the predator community would change, at least initially, with the advance of *D*. *v*. *virgifera*. *Diabrotica*-resistant *Bt* maize may potentially result in reduced abundance of general predators due to absence of their potential prey. Nevertheless, comparison with the effect of conventional pest management on such predators is necessary.

GM crops have significant potential for effective pest management while conserving beneficial natural enemies, including the diversity of generalist parasitoids and predators [28,60]. However, implementation of any GM event in most European countries has been hindered by fears of unpredictable environmental damage. The mistrust might be overcome by research such as this study but educational programs for growers and policy makers are also required. The deployment of GM maize MON88017, expressing the Cry3Bb1 toxin is a promising strategy for controlling a new and dangerous pest that continues to spread in Europe in spite of insecticide treatments. The toleration of current GM maize to the herbicide glyphosate provides an additional advantage for growers. Our analyses showed mostly similarity of abundance and diversity of above-ground arthropods in maize with the same genetic background, for both *Bt* (MON88017) and non-*Bt* (DK315) untreated or insecticide treated. Hybrids KIPOUS and PR38N86 showed some differences in species abundance relative to the *Bt* maize and its near-isogenic hybrid; this was probably due to the distinct hybrids’ characteristics. Since we did not detect any detrimental environmental effect of MON88017, this GM crop should be acceptable in the EU as the best alternative for curbing the spread of *D*. *v*. *virgifera*.

## Supporting Information

S1 Table Visual sampling of plant dwelling non-target arthropods. (XLS)

S2 Table Monitoring of parasitoids and predators on yellow sticky traps. (XLS)

S3 Table Cry3Bb1 measurement in plant tissues in 2009. (XLS)

S4 Table Cry3Bb1 measurement in plant tissues in 2010. (XLS)

S5 Table Cry3Bb1 measurement in plant tissues in 2011. (XLS)
